# Enhanced acetyl-CoA production is associated with increased triglyceride accumulation in the green alga *Chlorella desiccata*

**DOI:** 10.1093/jxb/erv166

**Published:** 2015-04-28

**Authors:** Omri Avidan, Alexander Brandis, Ilana Rogachev, Uri Pick

**Affiliations:** ^1^Department of Biological Chemistry, The Weizmann Institute of Science, Rehovot 76100, Israel; ^2^Biological Services Unit, The Weizmann Institute of Science, Rehovot 76100, Israel; ^3^Department of Plant and Environmental Sciences, The Weizmann Institute of Science, Rehovot 76100, Israel

**Keywords:** Acetyl-CoA, *Chlorella desiccata*, *Dunaliella tertiolecta*, green algae, pyruvate dehydrogenase, triglycerides.

## Abstract

Triglycerides (TAGs) from microalgae can be utilized as food supplements and for biodiesel production, but little is known about the regulation of their biosynthesis. This work aimed to test the relationship between acetyl-CoA (Ac-CoA) levels and TAG biosynthesis in green algae under nitrogen deprivation. A novel, highly sensitive liquid chromatography mass spectrometry (LC-MS/MS) technique enabled us to determine the levels of Ac-CoA, malonyl-CoA, and unacetylated (free) CoA in green microalgae. A comparative study of three algal species that differ in TAG accumulation levels shows that during N starvation, Ac-CoA levels rapidly rise, preceding TAG accumulation in all tested species. The levels of Ac-CoA in the high TAG accumulator *Chlorella desiccata* exceed the levels in the moderate TAG accumulators *Dunaliella tertiolecta* and *Chlamydomonas reinhardtii*. Similarly, malonyl-CoA and free CoA levels also increase, but to lower extents. Calculated cellular concentrations of Ac-CoA are far lower than reported *K*_mAc-CoA_ values of plastidic Ac-CoA carboxylase (ptACCase) in plants. Transcript level analysis of plastidic pyruvate dehydrogenase (ptPDH), the major chloroplastic Ac-CoA producer, revealed rapid induction in parallel with Ac-CoA accumulation in *C. desiccata*, but not in *D. tertiolecta* or *C. reinhardtii*. It is proposed that the capacity to accumulate high TAG levels in green algae critically depends on their ability to divert carbon flow towards Ac-CoA. This requires elevation of the chloroplastic CoA pool level and enhancement of Ac-CoA biosynthesis. These conclusions may have important implications for future genetic manipulation to enhance TAG biosynthesis in green algae.

## Highlight

Enhanced acetyl-CoA production, revealed by novel LC-MS/MS-based measurement and upregulation of plastidic pyruvate dehydrogenase, is associated with accumulation of triglycerides in the TAG-accumulating alga *Chlorella desiccata*.

## Introduction

Green microalgae are considered a major potential source of enriched nutritional supplements for human, animals, and fish, as well as for biomass and biodiesel production (Cardozo *et al.*, 2007; Harwood and Guschina, 2009). Intensive research is currently focused on characterizing and controlling the biosynthesis and accumulation of high-value storage products such as starch, triglyceride (TAG), carotenoids, and poly-unsaturated fatty acids (Napier, 2007; Hu *et al.*, 2008; Work *et al.*, 2010; Msanne *et al.*, 2012). However, current knowledge regarding the delicate regulation of carbon flow within cells and particularly in plastids is significantly lacking.

Numerous attempts to manipulate and control the tunnelling of carbon have been conducted in recent years, most of which are attempts to increase carbohydrates and lipid contents in oleaginous species through the expression or downregulation of various plastid-localized rate-limiting enzymes, e.g. coenzyme A carboxylase (ACCase) or diacylglycerol acyl transferase (DGAT). However, these attempts were mostly unsuccessful, probably due to the poor understanding of plastid regulatory mechanisms that control carbon flow in microalgae (Roesler *et al.*, 1997; Sasaki *et al.*, 1997; Bouvier-Navé *et al.*, 2000; Madoka *et al.*, 2002; Lardizabal *et al.*, 2008; Li *et al.*, 2010; Andre *et al.*, 2012). Interestingly, several recent studies have suggested a link between the rate of fatty acid (FA) and TAG biosynthesis to the availability of specific carbon precursors. It was demonstrated that increased carbon supply, either through the use of starch-less mutants, an exogenous acetate boost, or free-FA supplementation, strongly enhances TAG accumulation in several photosynthetic species (Goodson *et al.*, 2011; Siaut *et al.*, 2011; Fan *et al.*, 2012; Ramanan *et al.*, 2013). Also, carbon flux analyses have indicated that in most oil-accumulating plants the major metabolic control is exerted at the level of FA biosynthesis and includes several enzymatic stages (reviewed in Guschina *et al.*, 2014). An interesting comparison between oil palm and date palm revealed that TAG accumulation is mainly controlled by pyruvate supply and the consequent FA synthesis rate rather than acyl assembly into TAG (Bourgis *et al.*, 2011). Taken together, these studies imply that the extent of TAG synthesis in oleaginous species is limited by the rate of carbon flux towards plastids rather than by the level of expression of specific rate-limiting biosynthetic enzymes.

Curiously, while it is generally accepted that the main precursor for *de novo* FA biosynthesis in chloroplasts of photosynthetic species is acetyl coenzyme A (Ac-CoA), its production capacity has been largely ignored whereas its conversion to malonyl-CoA through the plastidic ACCase (ptACCase) was widely accepted as a major bottleneck for FA and TAG biosynthesis (Sasaki *et al.*, 1997; Davis *et al.*, 2000; Klaus *et al.*, 2004; Sasaki and Nagano, 2004; Huerlimann and Heimann, 2013). As a central metabolite, Ac-CoA participates not only in FA biosynthesis but also in mitochondrial respiration, isoprenoid biosynthesis, malonyl-CoA-derived metabolism, and in various acetylation reactions. These metabolic pathways take place in different subcellular compartments, involving distinct and non-exchangeable Ac-CoA pools, which complicate the analysis and understanding of their regulation (Nikolau *et al.*, 2000; Fatland *et al.*, 2005; Cai *et al.*, 2011). Nevertheless, the current techniques for detecting and quantifying CoA derivatives are lacking sensitivity and specificity and are limited to animal and plant tissues only. Only a few quantitative estimations of short-chain CoA derivatives in plant chloroplasts, seeds, or leaves have been reported, none of which suggested a link between the abundance of short CoA species and the rate of FA biosynthesis (Post-Beittenmiller *et al.*, 1992; Tumaney *et al.*, 2004; Hayashi and Satoh, 2006; Minkler *et al.*, 2006, 2008; Perera *et al.*, 2009).

In this research we aimed to test whether the availability of carbon precursors in the form of Ac-CoA may limit TAG accumulation in green microalgae. We developed a novel, highly sensitive liquid chromatography mass spectrometry (LC-MS/MS)-based technique that has provided the first ever reported data of Ac-CoA concentrations in green microalgae. We show that the level of Ac-CoA varies in a time-depended manner following nitrogen starvation, preceding TAG accumulation in *Chlorella desiccata*, *Dunaliella tertiolecta*, and *Chlamydomonas reinhardtii*.

## Materials and methods

### Algal strains and cultivation conditions

*Dunaliella tertiolecta* was obtained from the culture collection of Dr W. H. Thomas (La Jolla, CA, USA); *Chlamydomonas reinhardtii cw15* was obtained from Prof. A. Danon (Department of Plant Sciences at the Weizmann Institute, Rehovot, Israel) and *Chlorella desiccata* (UTEXID LB2437) was obtained from The Algae Culture Collection at the University of Texas at Austin, USA.

Cells were grown under continuous illumination (400 µmol m^–2^ s^–1^) in either artificial sea water (ASW) medium (*C. desiccata*,), TAP medium (*cw15*), or in 2M NaCl *Dunaliella* medium as previously described (Zalogin and Pick, 2014). In order to induce TAG accumulation, mid-log-phase cells were washed and transferred to a nitrogen-depleted (–N) medium at the following initial concentrations: *C. desiccata*, 2×10^7^ cells ml^–1^; *D. tertiolecta*, 2×10^6^ cells ml^–1^; and *cw15*, 1.5×10^6^ cells ml^–1^.

### TAG and starch analysis

Two methods were used for TAG quantification: Nile red fluorescence enhancement in live cells and thin-layer chromatography (TLC) in cell extract (Zalogin and Pick, 2014). Nile red was added to live cells in fresh growth medium (*C. desiccata*, 5×10^6^ cells ml^–1^; *D. tertiolecta*,1×10^6^ cells ml^–1^; *cw15*, 1×10^6^ cells ml^–1^) at a final concentration of 1 µM and measured after 3min through excitation at 488nm and emission at 580nm in a Cary Eclipse Spectrophotometer (Varian, Australia Pty-Ltd). For TLC analysis, lipids were extracted by the following method: pellets of 1–5×10^7^ cells were suspended with 200 μl of DMSO, heated to 70°C for 5min, mixed (vortex) with 3ml MeOH, and left for 12h at 4°C. Cell pellets were collected by centrifugation and saved for starch analysis, while the remaining supernatant was mixed vigorously with 3ml diethylether, 3ml *N*-hexane, and 3ml double-distilled water (DDW) before centrifugation for 5min at 4000rpm. The upper *N*-hexane phase was separated and evaporated in a desiccator; dried lipids were then re-suspended with 200 μl chloroform and kept at –20°C. 1–2 µl were applied to TLC silica-gel plates (5×7.5cm, 60 F254; Merck, Darmstadt, Germany) and developed in a closed jar in a mixture of *N*-hexane:diethylether:acetic acid (85:15:1, v/v/v). Lipid spots were visualized by 5min incubation in iodine vapour. The plate was scanned using an Image Scanner III, Epson ExpressionTM 10000 XL using scanning software LabScan™ 6.0 (Powered by Melanie, Swiss Institute of Bioinformatics). TAGs were quantified by densitometry software ImageQuant™ TL relative to different amounts of Triolein standards.

Starch content was determined by two methods. For iodine absorption, cell pellets of methanol-extracted cells and potato starch standards were supplemented with 200 µl of 70% ethanol, 400 µl DDW, and 400 µl of 2N NaOH. The samples were mixed and incubated for 2.5h at 25°C. Following that, 400 µl 2N HCl, 1ml 0.5M Na-acetate pH 4.8, and 7ml of DDW were added and mixed. Finally, 200 µl of the iodine reagent (1% KI + 0.1% I_2_) was added and mixed right before the absorption was read at 680nm. For the starch assay kit, 10 million cells were used, as described in the reagent protocol (Sigma Aldrich, SA-20).

### CoA analysis

#### Extract preparation

Fixed numbers of control and N-starved cells (*C. desiccata*, 5×10^9^; *D. tertiolecta* and *cw15*, 5×10^8^) were taken periodically and quenched with 3ml of cold (–20°C) acetonitryl/isopropanol (3:1). For extraction, pellets were mixed with glass beads (0.4 μm) and ground for 4min using a conical polypropylene tissue grinder, followed by addition of 1ml 0.1M KH_2_PO_4_ (pH 6.7) and kept at –80°C. For purification, we used a similar technique as described by Minkler *et al.* (2008), using self-prepared SPE columns of 2-(2-pyridyl) ethyl functionalized silica gel (300mg per column). Internal standards of ^13^C_2_-Ac-CoA or ^13^C_3_-malonyl-CoA (Sigma-Aldrich) (400ng each) were added just before the SPE purification and used for recovery normalization. Samples were then eluted with 7ml of methanol/250mM ammonium formate (4:1, pH 7) into glass tubes, evaporated under a stream of N_2_ for 3 hours and further lyophilized for 12–15h. Prior to injection, samples were re-suspended in 100 µl running buffer (10mM ammonium acetate, 5mM ammonium bicarbonate, pH 7) and centrifuged for 10min at 15 000rpm (4°C) before injection into measuring vials.

#### LC-MS/MS analysis

The LC-MS/MS instrument consisted of an Acquity I-class UPLC system (Waters) and Xevo TQ-S triple quadrupole mass spectrometer (Waters) equipped with an electrospray ion source and operated in positive ion mode for analysis of CoA and its acyls. Data acquisition and analysis were performed using MassLynx and TargetLynx software (v.4.1, Waters). Chromatographic separation was performed using a 100×2.1-mm i.d., 1.7-µm UPLC Kinetex XB-C18 column equipped with 2.1-mm i.d. SecurityGuard ULTRA C18 cartridge (both Phenomenex) with mobile phases A (10mM ammonium acetate and 5mM ammonium hydrocarbonate buffer, pH 7.0, adjusted with 10% acetic acid) and B (acetonitrile) at a flow rate of 0.3ml min^–1^ and column temperature 25°C. The gradient was as follows: 0–5.5min, linear increase 0–25% B, then 5.5–6.0min, linear increase till 100% B, 6.0–7.0min, hold at 100% B, 7.0–7.5min, back to 0% B, and equilibration at 0% B for 2.5min. Samples kept at 4°C were automatically injected in a volume of 3 μl.

For mass spectrometry, argon was used as the collision gas at a flow rate of 0.25ml min^–1^. The capillary voltage was set to 1.50kV, source temperature 150°C, desolvation temperature 350°C, and desolvation gas flow 650 l min^–1^. Analytes were detected using multiple reaction monitoring (MRM) applying the parameters listed below (Table 1).

**Table 1.** Parameters used for MRM detection of short acyl-CoA in samples

Compound	Retention time (min)	Transition	Cone (V)	CE (eV)
CoA	2.75	768.0 > 261.2	30	35
		768.5 > 428.0	30	25
Ac-CoA	3.76	810.0 > 303.1	30	35
		810.0 > 428.0	30	30
^13^C_2_-Ac-CoA	3.74	812.0 > 305.1	30	29
		812.0 > 428.0	30	22
Malonyl-CoA	2.29	854.1 > 245.3	30	30
		854.1 > 303.2	30	40
		854.1 > 347.2	30	30
		854.1 > 428.0	30	27
^13^C_3_-malonyl CoA	2.27	857.2 > 248.0	30	30
		857.2 > 305.3	30	40
		857.2 > 350.3	30	30
		857.2 > 428.0	30	27

The parameters Cone Voltage (Volts, V) and Collision Energy (CE) (eV) are used separately for each putative analyte to provide optimal parent and daughter ions for each compound in order to achieve the highest signal intensity for the chosen transition.

Quantification of compounds was done against external calibration curves, prepared by comparing the ratios of MRM peak areas of analyte to peak area of internal standard (P_analyte_/P_IS_). The same amount of internal standard was used for the preparation of both biological samples and calibration samples. For Ac-CoA and malonyl-CoA, their corresponding ^13^C analogues were used as internal standards. As there was no stable isotope-labelled CoA available, we used deuterated CoA as an internal standard for CoA quantification after checking that recoveries of CoA and Ac-CoA on the SPE cartridge were similar. The measurement ranges of Ac-CoA, malonyl-CoA, and CoA were 1–10 μg ml^–1^.

### Gene expression analysis

For RNA purification, collected samples were treated with TRI reagent according to the manufacturer’s protocol (Molecular Research Centre). Complementary DNA was synthesized using a qScript cDNA Synthesis Kit (Quanta) with 0.7 µg of purified RNA. *PDH-E1α* gene expression was determined by Real-Time PCR (qPCR) using PerfeCTa SYBR Green FasMix ROX (Quanta) with the following set of primers: *C. desiccata* (gb. KP293896), forward 5′-GCGTTCCAAATCGCATACAA and reverse 5′-GTTGCAAGTACCATCCCCAAA; *D. tertiolecta* (gb. EG591709), forward 5′-TCTCCGACAAGCACAACTTCT and reverse 5′-CAAAGAAGGAGCAGGTCACAG; *cw15* (jgi.155587), forward 5′-TCCGTGACCTGCTCCTTCTT and reverse 5′-GTAGAGCGCGGCCATGTT; *PFL1* (jgi. 146801), forward 5′-CGTTGGACTATGAGGAGGTCA and reverse 5′-CCGCTCGTAGTCGTACTTGTC. The level of expression was normalized according to selected endogenous genes, as follows: *C. desiccata*, *ACTIN* (gb.KP293895; the expression level of this actin gene was found to be the most stable and changed less than other genes tested under N deprivation in this species), forward 5′-CGCGACATCAAGGAGAAGCT and reverse 5′-TCTGAAGGGTGGAGGAAGCA; *D. tertiolecta*, *18S* (gb.EF473729; Davidi *et al.*, 2014), forward 5′-CGCGCTACACTGATGCATTC and reverse 5′-GACTCGCGCTTACTAGGCAT; *cw15*, Receptor of activated protein kinase C (*CBLP*: jgi.164254; Chiung-Wen *et al.*, 2005), forward 5′-CTCCATCAAGATCTGGGACCT and reverse 5′-TTCTTGCTGGTGATGTTGAACT.

## Results

In order to find out if Ac-CoA production and availability play a role in controlling TAG accumulation in green algae, we selected three green algae species that differ in their ability to accumulate starch or TAG and followed the changes in levels of Ac-CoA, malonyl-CoA, and free CoA during N starvation, which induces TAG accumulation. The first alga, *C. desiccata*, is a marine species which rapidly produces high TAG and low starch levels and thus serves as a TAG-accumulation model in our laboratory (Zalogin and Pick, 2014). The second, *D. tertiolecta*, is a halotolerant species that accumulates high starch and moderate TAG levels and has been extensively studied (Ben-Amotz *et al.*, 2009; Rismani-Yazdi *et al.*, 2011). The third is the well studied *C. reinhardtii* (*cw15*), which is grown mixotrophically and accumulates moderate levels of starch and TAG. If Ac-CoA production does limit TAG accumulation, then its levels are expected to increase before major TAG biosynthesis, correlating with the TAG-accumulation capacity of each alga.

### Differential accumulation of storage products following N starvation

**Fig. 1.**
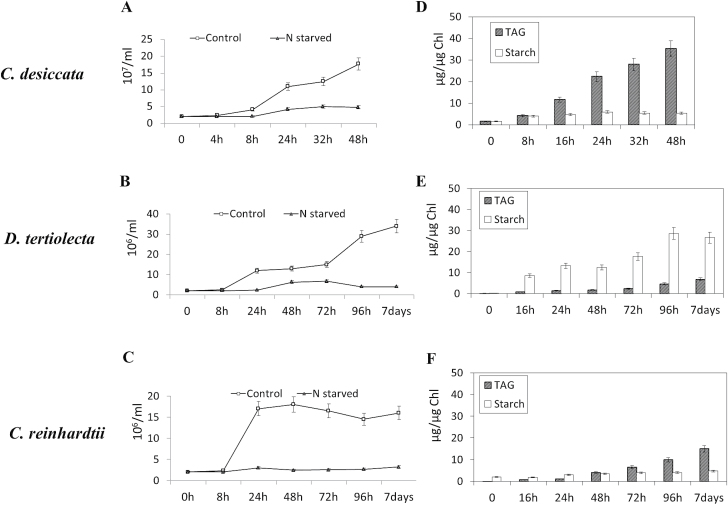
Culture growth, and TAG and starch accumulation following N starvation. Cell counts (A, B, C) and TAG and starch contents (D, E, F) were measured at the time points indicated following the onset of N starvation and normalized to Chl content. (A, D) *C. desiccata*; (B, E) *D. tertiolecta*; (C, F) *C. reinhardtii* (*cw15*). Data are mean ± SD of three independent experiments.

A comparison of starch and TAG accumulation under N starvation between the three green microalgae species is shown in Fig. 1. Culture growth in all three species was retarded under N starvation, but in *C. desiccata* and *D. tertiolecta* continued for one or two cell divisions (Fig. 1, A–C). The marine *C. desiccata* synthesized low levels of starch, which already saturates after 8h, and high levels of TAG, which continually accumulates for 48h, reaching over 30 µg µg^–1^ chlorophyll (Chl) (Fig. 1D). The halotolerant *D. tertiolecta* accumulated high levels of starch in two kinetic phases, between 0 and 24h and between 48 and 96h, and moderate levels of TAG, which accumulated mostly after 72h in correlation with the second phase of starch accumulation (Fig. 1E). The third species, *C. reinhardtii* (*cw15*), gradually accumulated moderate levels of TAG and starch (Fig. 1F). It is evident that TAG accumulation in *C. desiccata* is both faster and reaches much higher levels in comparison to *D. tertiolecta* and *cw15*.

### Variations in the levels of Ac-CoA, malonyl-CoA, and free CoA during N starvation

**Fig. 2.**
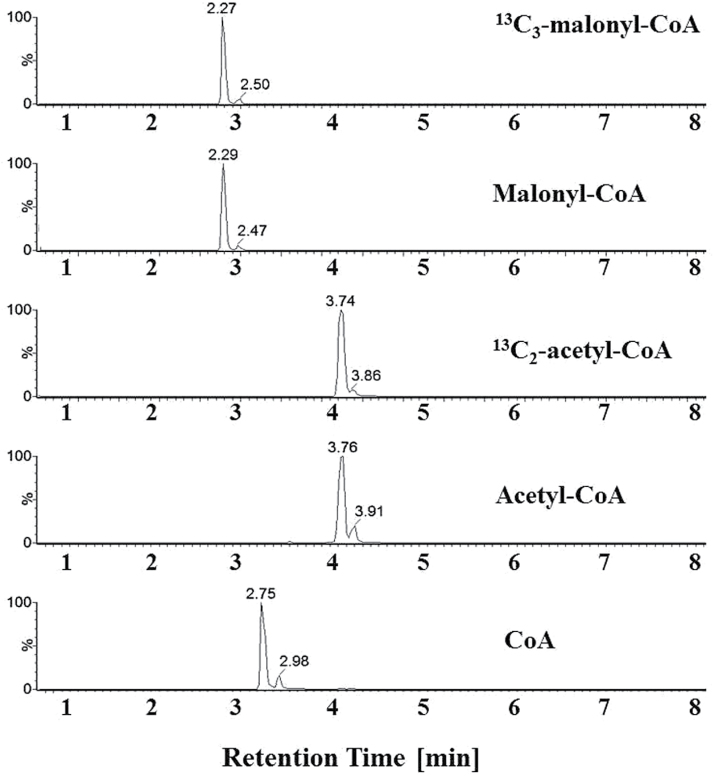
MRM chromatograms of acyl-CoA standards. Ac-CoA, malonyl-CoA, and free CoA were used for system calibration, whereas ^13^C_2_-Ac-CoA and ^13^C_3_-malonyl-CoA were utilized as internal standards for quantitative estimation as described in the Materials and Methods.

In order to learn if Ac-CoA levels change during the early stages of TAG induction in green algae, we developed a protocol for sensitive, accurate and time-saving determination of short-chain CoA derivatives. The preparation of cell extracts included quenching, grinding, and extraction in cold organic solvent (minimizing sample handling) and purification on a 2-(2-pyridyl) ethyl functionalized silica gel column. Extract samples were injected into a 1.7-µm UPLC Kinetex XB-C18 column and eluted in an ammonium acetate/acetonitrile gradient at pH 7.0. Mass spectrometry analysis was performed and analytes were detected using MRM and quantified using external calibration standards. ^13^C-analogues of Ac-CoA and malonyl-CoA were used for CoA quantification (see details in Materials and Methods). The average recoveries obtained for Ac-CoA, malonyl-CoA, and free CoA were 88%, 65%, and 50%, respectively, much higher than previously reported (Perera *et al.*, 2009). Fig. 2 shows traces of standards that were utilized, demonstrating the shortened retention time achieved by this method (10min per sample).

**Fig. 3.**
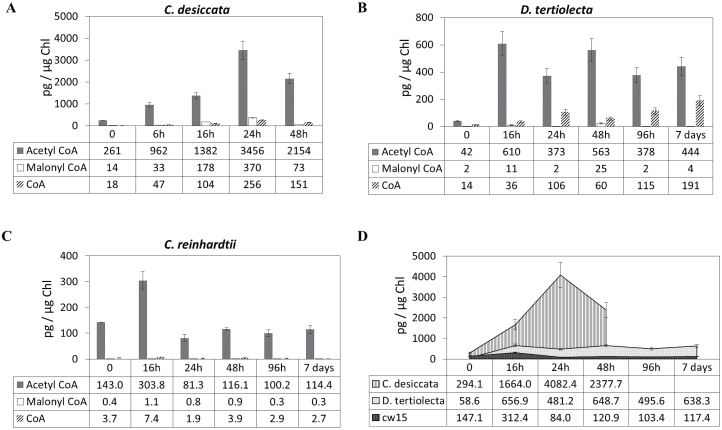
Variations in levels of Ac-CoA, malonyl-CoA, and free CoA following N starvation. Samples were taken periodically, quenched, processed, and purified before injection for LC-MS/MS analysis. Values were normalized to Chl content. (A) *C. desiccata*; (B) *D. tertiolecta*; (C) *C. reinhardtii cw15*. (D) Total CoA pool: the sum of acetyl, malonyl, and free CoAs at each time point. Data are mean ± SD of four independent experiments.

As seen in Fig. 3, N starvation induced dramatic variations in the level of Ac-CoA in all three species, but to varying degrees. In the high-TAG accumulator, *C. desiccata*, Ac-CoA levels already rose rapidly after 6h and continued to increase until 24h, reaching over 10-fold of the resting levels, followed by a slight decrease at 48h (Fig. 3A). In *D. tertiolecta* and *cw15*, Ac-CoA levels also rose rapidly, already reaching maxima after 16h of N starvation, but the maximal levels were only 10–20% of the levels in *C. desiccata* (Fig. 3B–C). The high sensitivity of our method enabled us also to determine and quantify the levels of free CoA and malonyl-CoA, the substrate for Ac-CoA synthesis and first dedicated precursor for *de novo* FA synthesis and elongation, respectively.

Generally, the levels of free CoA and malonyl-CoA were much lower than that of Ac-CoA in all three species at all time-points, indicating that Ac-CoA is by far the major CoA species in cells, in agreement with previous results in plants (Tumaney *et al.*, 2004; Hayashi and Satoh, 2006). In *C. desiccata*, both free CoA and malonyl-CoA increased in parallel with Ac-CoA, reflecting the large increase in total CoA pool size (Fig. 3D). In *D. tertiolecta* and *cw15*, the levels of both free and malonyl-CoA also rose with time, roughly in parallel with the rise of Ac-CoA, but to much lower levels than in *C. desiccata* (especially *cw15*). It is noteworthy that the levels of free CoA in *D. tertiolecta* continually rose with time, comprising about a third of the total CoA pool after 7 days (Fig. 3B).

The large increase in Ac-CoA during N starvation, especially in the TAG accumulator *C. desiccata,* suggests a massive synthesis of this coenzyme that increases the total CoA pool together with enhanced conversion of CoA to Ac-CoA within the chloroplast.

### Is the mRNA expression of ptPDH-E1α correlated with Ac-CoA accumulation?

**Fig. 4.**
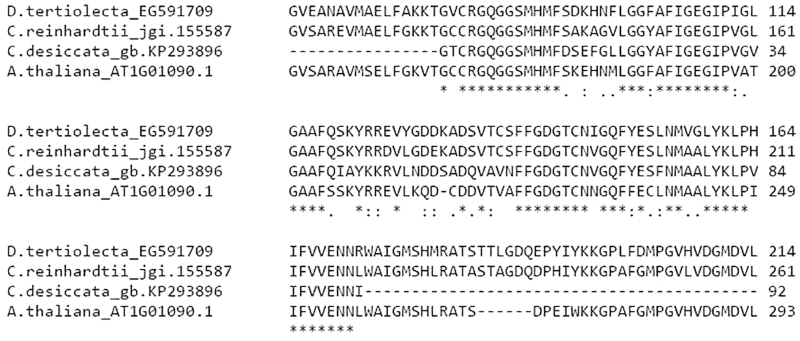
Alignment of ptPDH-E1α amino acid partial sequences.

Since the major enzyme producing Ac-CoA in plant chloroplasts is plastidic pyruvate dehydrogenase (ptPDH) (Camp and Randall, 1985; Ke *et al.*, 2000; Rawsthorne, 2002; Tovar-Mendez *et al.*, 2003), it is the primary target suspected to explain the enhanced production of Ac-CoA in *C. desiccata* during N starvation. In order to test if this enzyme was being upregulated during N starvation, we followed the transcription levels of the ptPHD-E1α subunit, known to regulate the assembly of the PDH complex (Johnston *et al.*, 2000). Because the genome of *C. desiccata* is neither sequenced nor annotated, the ptPDH-E1α gene had to be first sequenced using the Rapid Amplification Chain Elongation (RACE) procedure and compared with the homologous genes from *D. tertiolecta*, *C. reinhardtii*, and *Arabidopsis thaliana* (Fig. 4).

**Fig. 5.**
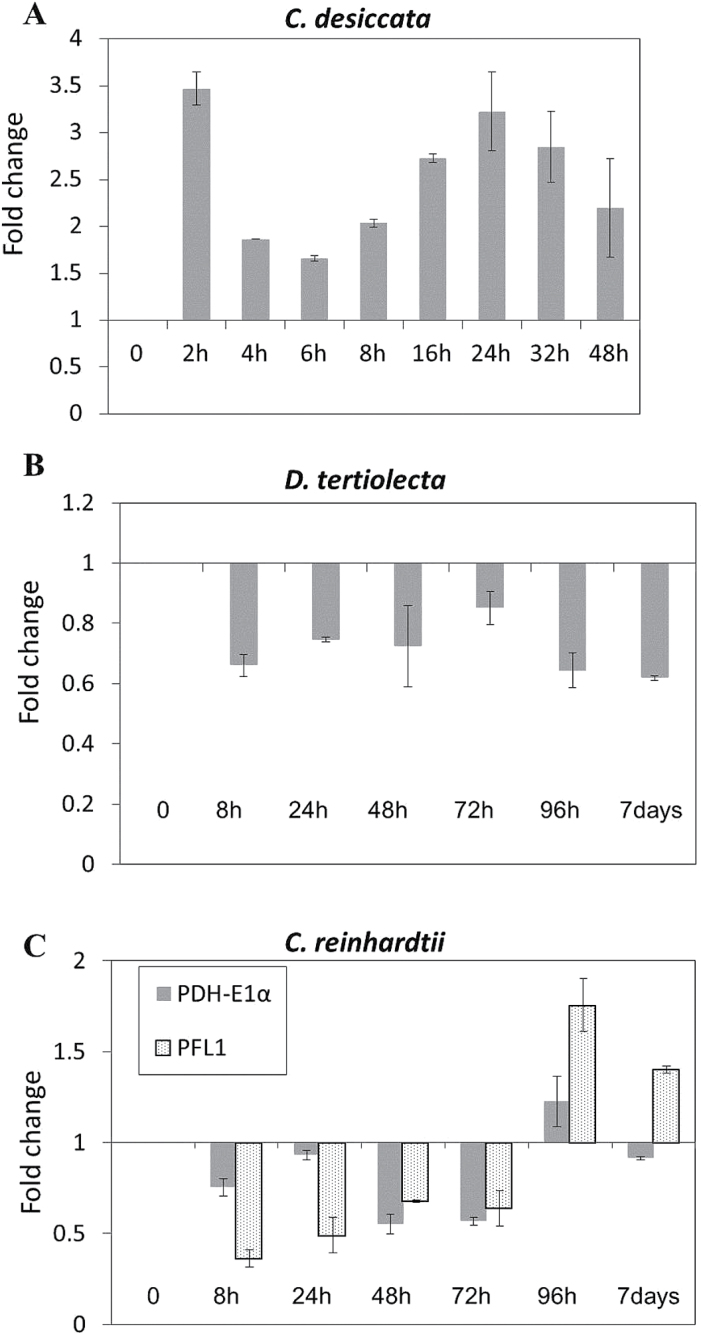
mRNA expression levels of plastidic PDH-E1α. Cultures were grown under continuous high light (400 µmol m^–2^ s^–1^) and induced by N starvation. Samples taken at the time points indicated were processed for RNA purification and cDNA synthesis using oligo-DT primers. The qPCR measurements were normalized to selected endogenous genes and presented as a fold-change relative to non-induced cells. (A) *C. desiccata*; (B) *D. tertiolecta*; (C) *C. reinhardtii* (*cw15*).

Since the level of Ac-CoA already rises within the first 6h of N starvation in *C. desiccata*, we tested shorter time intervals of ptPDH-E1α mRNA expression in this species. As seen in Fig. 5, the transcription pattern of PDH-E1α varied remarkably between the species. In *C. desiccata*, it was already transiently induced within 2h (3.5-fold increase) followed by a rapid decrease and again reactivation, peaking at 24h of N starvation. Conversely, in *D. tertiolecta* and *cw15*, PDH-E1α was downregulated by ~20–40% during N starvation, except for a late upregulation at 96h and 7 days in *cw15.* Since *cw15* possesses several alternative bacterial-like CoA producers, it was of interest to test if such enzymes may substitute for PDH in this species. One such Ac-CoA producer is the plastidic pyruvate formate-lyase (PFL1), known to play a major role in the life cycle of *cw15* (Hemschemeier *et al.*, 2008; Catalanotti *et al.*, 2012, 2013). However, as can be seen in Fig. 5C, the transcription of PFL1 followed a similar time-course to PDH-E1α, namely suppression followed by a small induction, in line with a previous report (Blaby *et al.*, 2013). Moreover, the late upregulation of PDH-E1α and PFL1 was not correlated with the fast elevation in Ac-CoA levels detected in this species during N starvation. Taken together, it was only in *C. desiccata* that we observed a correlation between enhanced ptPDH-E1α transcription and the increase in Ac-CoA levels, suggesting that upregulation of ptPDH may be responsible for rapid production of Ac-CoA in chloroplasts of this species during N starvation.

## Discussion

The small number of quantitative determinations of short CoA derivatives in plant systems in general and microalgae in particular is largely due to technical difficulties in extraction and analysis of these central metabolites. The improved method reported here now makes it possible to determine and quantify Ac-CoA, malonyl-CoA, and free CoA in plants, microalgae, and many other organisms. Since there are hardly any reports on the levels of short CoA derivatives in relation to the regulation of lipid biosynthesis in microalgae, it is interesting to compare our findings with what is known in plants. The levels of CoA derivatives in plants have been determined in chloroplasts of leaves and in oil seeds, which differ quite remarkably. Unicellular green algae resemble plant leaves in that the FAs are made in chloroplasts and assembled in the endoplasmatic reticulum, but they also resemble oil seeds in the massive production of TAG which accumulates in lipid droplets (Liu and Benning, 2013).

An early analysis of Ac-CoA and malonyl-CoA in spinach and pea chloroplasts revealed that most of the CoA pool in leaves is concentrated in the chloroplasts, that the major CoA species is Ac-CoA, similar to our findings, and that the calculated concentrations of Ac-CoA are far lower than the concentrations determined previously in bacteria and animal tissues (Post-Beittenmiller *et al.*, 1992). Notably, similar concentrations of Ac-CoA were measured in light-adapted and dark-adapted chloroplasts irrespective of the light-stimulation of FA biosynthesis, indicating that production of Ac-CoA is not correlated with FA biosynthesis. Conversely, in rice seeds, it was found that both Ac-CoA and malonyl-CoA levels dramatically increase during seed maturation in association with the accumulation of TAG (Hayashi and Satoh, 2006).

The rapid increase in Ac-CoA levels during N starvation was observed in all green algae species tested here. The maximal level of Ac-CoA always preceded the maximal TAG level. Taken together with the finding that the high TAG-accumulating species *C. desiccata* accumulated substantially higher levels of Ac-CoA, these results imply that the increase in level of Ac-CoA is closely associated with the capacity to accumulate high TAG levels. This conclusion is consistent with similar finding in maturing oil seeds (Hayashi and Satoh, 2006). Accordingly, these results suggest that Ac-CoA concentration may be a rate-limiting parameter in TAG biosynthesis in green algae.

The calculated average cellular concentrations of Ac-CoA that we determined in green algae range from about 1 to 15 μM (Table 2). Based on the studies of plant leaves, it may be assumed that most of the Ac-CoA is concentrated in the chloroplast, whose volume is approximately half the cell volume, resulting in estimated concentrations within the chloroplast of at most 2–30 μM. These calculated concentrations are even lower than the estimated values in plant chloroplasts of 30–65 μM (Post-Beittenmiller *et al.*, 1992; Bao *et al.*, 2000; Tumaney *et al.*, 2004; Hayashi and Satoh, 2006).

**Table 2.** Calculated cellular concentrations of Ac-CoA, malonyl-CoA, and free CoA

	Alga					
Parameter	*D. tertiolecta*		*C. desiccata*		*cw15*	
Time (h)	0	48	0	24	0	16
Volume (fl cell^–1^)	110	160	15	22	100	125
[Ac-CoA] μM	0.7	3.9	2.9	14.1	4.4	5.5
[Mal-CoA] μM	0.05	0. 17	0.17	1.45	0.01	0.02
[CoA] μM	0.32	0.44	0.20	1.13	0.10	0.15

The data acquired by LC-MS/MS and measured cellular volumes were used to calculate cellular concentrations of CoA species at non-induced (0) and peak levels reached under N-deprived conditions in each species of alga.

To find out if the concentration range of 1–15 μM (or 2–30 μM) has kinetic significance, it should be compared to the *K*_m_ value of ptACCase for Ac-CoA. Reported values of the *K*_m_ for Ac-CoA of ptACCase exceed 100 μM (Nikolau *et al.*, 1984; Post-Beittenmiller *et al.*, 1992; Alban *et al.*, 1994; Nikolau *et al.*, 2003; Tumaney *et al.*, 2004; Feria Bourrellier *et al.*, 2010), far above the calculated values in our study. Accordingly, any increase in Ac-CoA should result in a proportional increase in the rate of conversion of Ac-CoA to malonyl-CoA, consistent with the idea that low concentrations of Ac-CoA limit TAG biosynthesis.

The large increase in Ac-CoA during N starvation, particularly in *C. desiccata*, may result from accelerated biosynthesis of CoA and/or from accelerated formation of Ac-CoA from free CoA and pyruvate. The roughly parallel increase in levels of Ac-CoA, malonyl-CoA, and free CoA in *C. desiccata* and in the two other algae suggests that the major contributor to the increase in Ac-CoA concentrations is the change in pool size, resulting from accelerated biosynthesis of this coenzyme during N starvation.

The biosynthetic pathway of CoA is well established and its inhibition has been associated with growth retardation and impaired resistance to different stress conditions as well as to lipid storage (Rubio *et al.*, 2006, 2008). These findings combined with the increase in CoA pool size in oils seeds (Hayashi and Satoh, 2006) and in our work indicate the importance of the CoA pool size in regulation of the carbon flux towards enhanced FA and TAG biosynthesis under stress conditions.

Accelerated formation of Ac-CoA from free CoA (and pyruvate) in *C. desiccata* can be attributed to the rapid overexpression of ptPDH-E1α in this alga, as implied by the fast increase in mRNA transcript level (Fig. 5). More rigorous confirmation for upregulation of ptPDH by demonstration of an increase in protein level or of enzymatic activity could not be obtained because anti-ptPDH antibodies are not available and could not be produced in animal hosts; there is also no way to discriminate between mitochondrial PDH (mtPDH) and ptPDH activities in crude cell extracts, resulting in a very high background activity of mtPDH, which exceeds ptPDH activity in plants (Williams and Randall, 1979). However, there is circumstantial evidence that supports upregulation of ptPDH: it has recently been demonstrated that downregulation of ptPDH E1*α* in *C. reinhardtii* cultured under N deprivation and autotrophic conditions impairs triglyceride accumulation (Shtaida *et al.*, 2014) suggesting that it may be a critical enzyme for TAG biosynthesis in green algae. Also, the increase in the ratio [Ac-CoA]/[CoA], which peaks at 6h of N starvation in *C. desiccata* (Table 3), is consistent with this idea.

**Table 3.** Calculated ratios: [Ac-CoA]/[CoA] and [Mal-CoA]/[Ac-CoA]

	Alga		
Time –N	*D. tertiolecta*	*C. desiccata*	*C. reinhardtii*
	[Ac-CoA]/[CoA]		
0	3.0	14.4	38.7
6 h	–	20.5^a^	–
16 h	16.9^a^	13.3	41.0
24 h	3.5	13.5	42.8
48 h	9.4	14.3	29.8
96 h	3.3	–	34.6
7 days	2.3	–	42.4
	**[Mal-CoA]/[Ac-CoA]**		
0	0.048	0.05	0.003
6 h	–	0.04	–
16 h	0.018	0.13^a^	0.004
24 h	0.005	0.11	0.010^a^
48 h	0.045	0.07	0.008
96 h	0.005	–	0.003
7 days	0.009	–	0.003

^a^ Maximal metabolite concentration ratios.

The finding that ptPDH-E1α is not induced in *D. tertiolecta* and *cw15* (also PFL1), which accumulate a low TAG content, is in line with the high levels of free CoA and relatively low Ac-CoA levels observed in these species, respectively. The increase in the ratio [Ac-CoA]/[CoA] after 16h in *D. tertiolecta* may thus imply that another enzyme contributes to Ac-CoA production in this species, possibly in a separate compartment.

Similar to plant chloroplasts, our results confirm that the concentrations of free CoA and malonyl-CoA are far lower than the concentration of Ac-CoA in all three species, but different from seeds in which relatively high levels of malonyl-CoA were detected (Tumaney *et al.* 2004; Hayashi and Satoh, 2006). These findings may suggest that in N-deprived green algae, the kinetics of *de novo* FA biosynthesis is such that the utilization of malonyl-CoA is relatively faster than its formation via ptACCase.

The ratio [malonyl-CoA]/[Ac-CoA] can provide an indication of the relative ptACCase activity, although it is also influenced by the rate of Ac-CoA production and malonyl-CoA utilization. Nevertheless, this ratio in *C. desiccata* first increases from 0 to 16h and then decreases from 16 to 48h of N starvation (Table 3), implying a rapid activation of ptACCase followed by a subsequent activation of downstream enzymes leading to accelerated malonyl-CoA utilization towards FA biosynthesis. Interestingly, the peak [malonyl-CoA]/[Ac-CoA] ratio after 16h follows the peak of [Ac-CoA]/[CoA] at 6h, consistent with the idea that low Ac-CoA concentrations limit ACCase activity. In *D. tertiolecta* and *cw15*, however, the ratio [malonyl-CoA]/[Ac-CoA] yields substantially lower values, possibly reflecting lower ACCase activity, which is consistent with the relatively low Ac-CoA levels observed in these species.

**Fig. 6.**
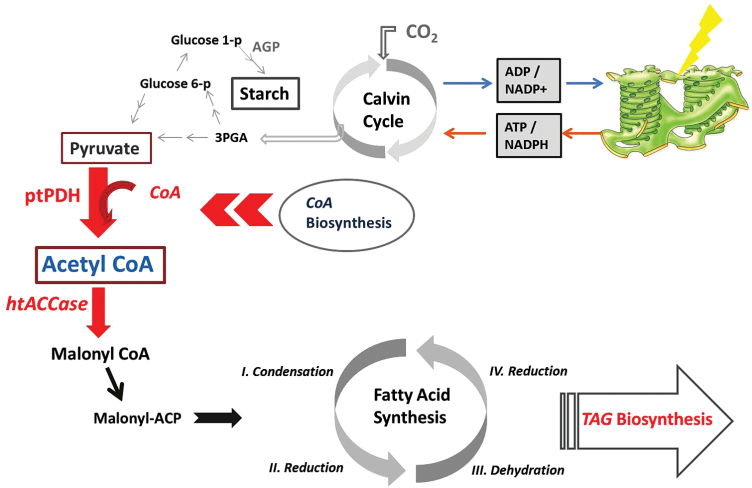
Schematic representation of the Ac-CoA-related metabolic pathway involved in FA and TAG biosynthesis following N starvation. Bold arrows signify reactions that are enhanced under N starvation. This figure is available in colour at *JXB* online.

A hypothetical scheme representing the metabolic pathway leading to TAG biosynthesis under N deprivation in *C. desiccata*, stressing the elevation of Ac-CoA, is depicted in Fig. 6.

In summary, the finding that during N starvation there is a large increase in Ac-CoA levels and that the largest increase is observed in *C. desiccata* suggests that the capacity to accumulate TAG in this oleaginous alga, and possibly other oleaginous species, critically depends on the ability to rapidly produce Ac-CoA in order to divert carbon flux towards FA and TAG biosynthesis. The finding that steady state Ac-CoA concentrations in chloroplasts seem to be far below the *K*_m_ of the ptACCase for this substrate implies that the low substrate concentration may act as a threshold to control the carbon flux towards TAG biosynthesis, which is an unorthodox mechanism of control. These results may have practical applications for future attempts to enhance lipid biosynthesis in microalgae, e.g. overexpression of rate-limiting enzymes in CoA biosynthesis in chloroplasts can lead to elevated levels of Ac-CoA and this in turn may enhance FA and TAG biosynthesis.

## Funding

This work was supported by the Alternative Energy Research Initiative Fund at the Weizmann Institute of Science and by the Minerva centre: Photosynthesis under stress.

## Abbreviations:

TAG: triglyceride
FA: fatty acid
Ac-CoA: acetyl coenzyme A
LC MS/MS: liquid chromatography mass spectrometry
ptACCase: plastidic coenzyme A carboxylase
ptPDH: plastidic pyruvate dehydrogenase
DGAT: diacylglycerol acyl transferase
TLC: thin-layer chromatography
DDW: double-distilled water
MRM: multiple reaction monitoring
Chl: chlorophyll.
